# Special considerations for studies of extracellular vesicles from parasitic helminths: A community‐led roadmap to increase rigour and reproducibility

**DOI:** 10.1002/jev2.12298

**Published:** 2023-01-05

**Authors:** Ruby White, Javier Sotillo, María Eugenia Ancarola, Anne Borup, Anders Toftegaard Boysen, Paul J. Brindley, Edit I. Buzás, Serena Cavallero, Sujittra Chaiyadet, Iain W. Chalmers, Marcela A. Cucher, Maude Dagenais, Chelsea N. Davis, Eileen Devaney, Maria A. Duque‐Correa, Ramon Marc Eichenberger, Santiago Fontenla, Thomas A. Gasan, Cornelis H. Hokke, Maja Kosanovic, Marije E. Kuipers, Thewarach Laha, Alex Loukas, Rick M. Maizels, Antonio Marcilla, Hynek Mazanec, Russell M. Morphew, Kyriaki Neophytou, Linh Thuy Nguyen, Esther Nolte‐‘t Hoen, Michael Povelones, Mark W. Robinson, Alicia Rojas, Irma Schabussova, Hermelijn H. Smits, Sivapong Sungpradit, Lucienne Tritten, Bradley Whitehead, Amin Zakeri, Peter Nejsum, Amy H. Buck, Karl F. Hoffmann

**Affiliations:** ^1^ The University of Edinburgh Institute of Immunology and Infection Research School of Biological Sciences Edinburgh UK; ^2^ Instituto de Salud Carlos III National Center for Microbiology Majadahonda Madrid Spain; ^3^ Department of Microbiology School of Medicine University of Buenos Aires Buenos Aires Argentina; ^4^ Institute of Research on Microbiology and Medical Parasitology (IMPaM UBA‐CONICET) University of Buenos Aires Buenos Aires Argentina; ^5^ Department of Clinical Medicine Aarhus University Aarhus Denmark; ^6^ George Washington University Microbiology Immunology & Tropical Medicine School of Medicine & Health Sciences Washington, D.C. USA; ^7^ ELKH‐SE Immune Proteogenomics Extracellular Vesicle Research Group Budapest Hungary; ^8^ HCEMM‐SU Extracellular Vesicle Research Group Budapest Hungary; ^9^ Department of Public health and infectious diseases Sapienza University of Rome Rome Italy; ^10^ Tropical Medicine Graduate Program Academic Affairs Faculty of Medicine Khon Kaen University Khon Kaen Thailand; ^11^ Aberystwyth University Institute of Biological Environmental & Rural Sciences (IBERS) Aberystwyth Ceredigion Wales UK; ^12^ McGill University Institute of Parasitology Ste‐Anne‐de‐Bellevue Quebec Canada; ^13^ University of Glasgow Institute of Biodiversity Animal Health and Comparative Medicine College of Medical Veterinary and Life Sciences Glasgow UK; ^14^ Wellcome Sanger Institute Parasites and Microbes Cambridge UK; ^15^ University of Zurich Institute of Parasitology Vetsuisse Faculty Zurich Switzerland; ^16^ Universidad de la República Facultad de Medicina Departamento de Genetica Montevideo Uruguay; ^17^ Queen's University Belfast School of Biological Sciences Belfast Northern Ireland UK; ^18^ Leiden University Medical Center Parasitology Leiden The Netherlands; ^19^ Institute for the Application of Nuclear Energy INEP University of Belgrade Belgrade Serbia; ^20^ Utrecht University Faculty of Veterinary Medicine Biomolecular Health Sciences Utrecht The Netherlands; ^21^ Department of Parasitology Faculty of Medicine Khon Kaen University Khon Kaen Thailand; ^22^ James Cook University Australian Institute of Tropical Health and Medicine Cairns Queensland Australia; ^23^ University of Glasgow Wellcome Centre for Integrative Parasitology Institute of Infection Immunity and Inflammation Glasgow UK; ^24^ Universitat de València Departamento Farmacia y Tecnología Farmacéutica y Parasitología Área de Parasitología Burjsassot Valencia Spain; ^25^ Faculty of Science University of South Bohemia České Budějovice Czech Republic; ^26^ Czech Republic Institute of Parasitology Biology Centre Czech Academy of Sciences České Budějovice Czech Republic; ^27^ Department of Biochemical Sciences Faculty of Pharmacy Charles University Prague Czech Republic; ^28^ University of Pennsylvania School of Veterinary Medicine Pathobiology Philadelphia Pennsylvania USA; ^29^ Laboratory of Helminthology Faculty of Microbiology University of Costa Rica San Pedro Montes de Oca Costa Rica; ^30^ Medical University of Vienna Institute of Specific Prophylaxis and Tropical Medicine Vienna Austria; ^31^ Department of Pre‐clinic and Applied Animal Science Faculty of Veterinary Science Mahidol University Nakhon Pathom Thailand; ^32^ Swiss Tropical and Public Health Institute Allschwil Switzerland; ^33^ University of Basel Basel Switzerland

**Keywords:** electron microscopy, EV guidelines, EV reporting, extracellular vesicles, helminths, parasites

## Abstract

Over the last decade, research interest in defining how extracellular vesicles (EVs) shape cross‐species communication has grown rapidly. Parasitic helminths, worm species found in the phyla Nematoda and Platyhelminthes, are well‐recognised manipulators of host immune function and physiology. Emerging evidence supports a role for helminth‐derived EVs in these processes and highlights EVs as an important participant in cross‐phylum communication. While the mammalian EV field is guided by a community‐agreed framework for studying EVs derived from model organisms or cell systems [e.g., Minimal Information for Studies of Extracellular Vesicles (MISEV)], the helminth community requires a supplementary set of principles due to the additional challenges that accompany working with such divergent organisms. These challenges include, but are not limited to, generating sufficient quantities of EVs for descriptive or functional studies, defining pan‐helminth EV markers, genetically modifying these organisms, and identifying rigorous methodologies for in vitro and in vivo studies. Here, we outline best practices for those investigating the biology of helminth‐derived EVs to complement the MISEV guidelines. We summarise community‐agreed standards for studying EVs derived from this broad set of non‐model organisms, raise awareness of issues associated with helminth EVs and provide future perspectives for how progress in the field will be achieved.

## 1 INTRODUCTION

Mammalian extracellular vesicles (EVs) were first discovered in reticulocytes and hypothesised to be vehicles for disposing of cellular material as they differentiate into erythrocytes (Johnstone et al., 1987). Not until the late 1990s when B lymphocytes and dendritic cells (DCs) were shown to release functional EVs, did the field begin to appreciate that EVs could have diverse biological roles (Raposo et al., 1996; Zitvogel et al., 1998). Although studies of mammalian systems paved the way for much of the current understanding of EV structure, cargo, and function, it is important to note that the discovery of multivesicular bodies (MVBs) and outer membrane vesicles (OMVs) in algae and plants, and bacteria, respectively (Bishop & Work, 1965; De, 1959; Jensen, 1965) had already introduced these excreted/secreted (E/S) cellular agents as important players in diverse biological systems. Despite the ubiquitous presence of EVs across archaea, eukarya and bacteria (Woith et al., 2019), our knowledge of EV functions remains incomplete. In the last decade alone it has become increasingly clear that EVs play an important role in inter‐ and intraspecies communication, including host‐parasite and parasite‐parasite interactions (Coakley et al., 2015; Drurey & Maizels, 2021).

Parasitic helminths (i.e., worms) have a considerable socioeconomic impact, infecting more than 1.5 billion people worldwide and imposing additional burdens on livestock systems, affecting animal welfare, food security, and sustainable agriculture (WHO, 2020). These pathogens can survive for years within their natural hosts, which represent an inhospitable environment, mainly due to their ability to modulate both the host's immune system and physiological state. Indeed, many helminth‐derived molecules have been identified that play roles in host immune modulation and, in recent years, EVs have been implicated in this process (Ryan et al., 2020). For example, EVs from different helminth species, packed with potential immune modulators interact with host cells and, upon uptake, modulate gene expression and affect cellular systems (Drurey & Maizels, 2021; Sánchez‐López et al., 2021). Some of the most compelling evidence in support of the essential role of helminth‐derived EVs during infection is their ability to prevent or reduce subsequent infections when used as vaccines (Chaiyadet et al., 2019; Coakley et al., 2017; Drurey et al., 2020; Mekonnen et al., 2020; Phumrattanaprapin et al., 2021; Shears et al., 2018; Trelis et al., 2016). Therefore, helminth‐derived EVs, and the biomolecules that they carry as cargo, represent attractive candidates for progressing novel intervention strategies in combating helminthiases.

**FIGURE 1:**
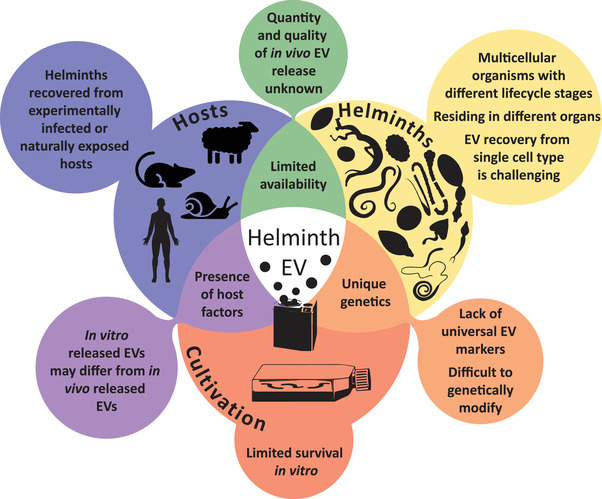
Collection of EVs derived from parasitic helminths is challenging and requires special considerations. These challenges/special considerations are segregated into three main criteria: acquisition from unrelated hosts (purple), diversity of metazoan helminths undergoing distinct lifecycles (yellow) and differing conditions required for in vitro cultivation (dark orange). Each of these components poses their own limitations/challenges (summarised in similar color extensions), but also can be synergistically affected by each other (summarised by overlapping parts of each circle). These considerations are specific for parasitic helminth EV investigators and are additional to the general challenges as summarized by MISEV 2018. Part of the figure was created with adjusted images from Servier Medical Art, provided by Servier, licensed under a Creative Commons Attribution 3.0 unported license.

Accordingly, a growing number of scientists have started to study EVs from parasitic helminths and, therefore, face the same challenges as other fields (e.g., the mammalian field) in standardising methodologies to ensure rigour and enable inter‐laboratory comparisons. The mammalian EV field addressed these issues in the 2018 MISEV guidelines (Théry et al., 2018). Where possible, this framework should be applied to parasitic helminth EV research. However, parasitic helminths are non‐model organisms of different sizes, host relationships, and life cycle transitions. Moreover, genomes of parasitic helminths are often different than those of their closely related models or free‐living organisms, adding intrinsic complexity (Blaxter & Koutsovoulos, 2015; Coghlan et al., 2019; Viney, 2018). Because of these differences, the study of parasitic helminth‐derived EVs presents additional challenges that require further consideration for adopting uniform guidelines (Figure 1).

**FIGURE 2:**
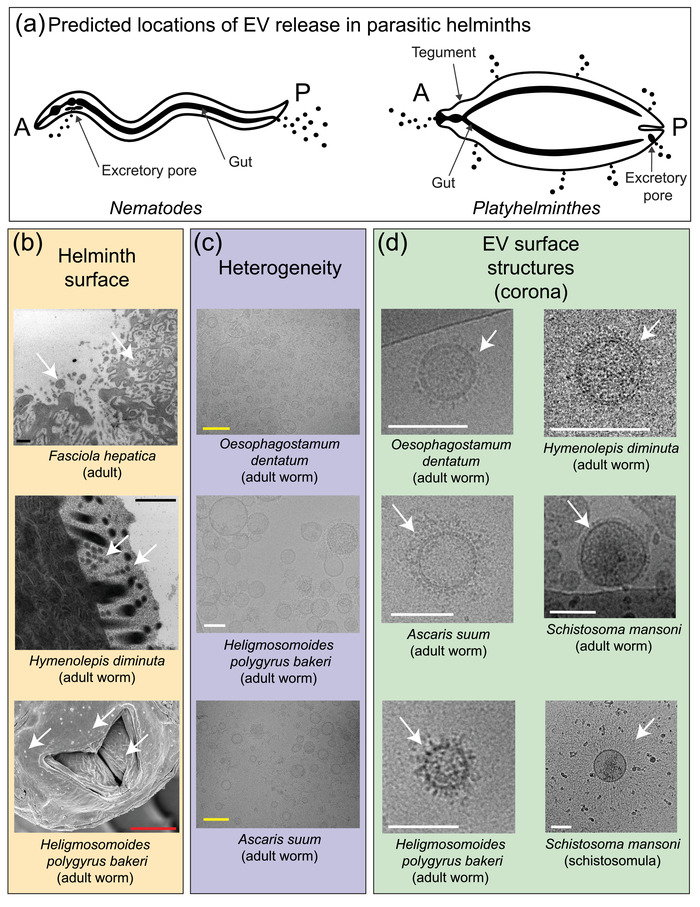
Parasitic helminths produce heterogenous populations of excreted/secreted EVs. (a) Pictorial representation of where EVs (black spheres) are released in illustrative nematode and platyhelminth species. A = anterior; P = posterior. In nematodes, EVs are likely released from the excretory pore and the gut (via the anus); in platyhelminthes, EVs are released from the tegumental surface, the gut (via the mouth) and the excretory pore. (b) Representative examples of EVs being released from the surface of adult *Fasciola hepatica* and *Hymenolepis diminuta* platyhelminths as assessed by TEM and presence of EV like structures on surface of *Heligmosomoides polygyrus bakeri* near the mouth, as assessed by SEM. EV diversity (e.g., size and morphology) is marked by white arrows; when parasitic worms are incubated in vitro, the conditioned culture medium will contain these (and other) EV populations. (c) Cryo‐TEM visualization of EV heterogeneity following separation from culture media for exemplar parasitic nematodes (*Oesophagostomum dentatum*, *Ascaris suum* and *H. polygyrus bakeri*). EVs originating from *O. dentatum*, *A. suum* and *H. polygyrus bakeri* culture supernatants were separated by ultracentrifugation (UC) followed by size exclusion chromatography (SEC). (d) Cryo‐TEM analyses detect corona‐like surface structures (white arrows) on separated EVs obtained from both parasitic nematodes (*O. dentatum*, *A. suum* and *H.polygyrus bakeri*) and platyhelminths (*S. mansoni*, adults and schistosomula, as well as *H. diminuta*). *O. dentatum* and *A. suum* EVs were separated by UC and SEC; *H. diminuta* EVs were separated by UC; *H. polygyrus bakeri* EVs were separated UC, followed by SEC; *S. mansoni* adult EVs were separated by UC, followed by purification through iodixanol density gradients; *S. mansoni* schistosomula EVs were separated by UC. Red scale bar = 5 μm, black scale bar = 500 nm, yellow scale bar = 250 nm and white scale bar = 100 nm.

‘Parasitic helminth’ is a broad term referring to a large assemblage of metazoans, contained within evolutionarily distinct phyla (Nematoda and Platyhelminthes). Importantly, each species is governed by discrete life cycles and host (one or more) interactions. Thus, working with helminth EVs can be challenging as most parasitic species cannot be maintained in culture for their whole life cycle, or under conditions that closely mimic the host environment(s). Therefore, obtaining EVs from helminth species relies on complicated and time‐consuming passage through experimentally infected animals or recovery of parasites from natural hosts; this introduces significant constraints on quantities of EVs that can be acquired. In addition, some species or life cycle stages may be particularly difficult to recover due to their location in the host or limited survival during in vitro culture. Complexity (i.e., contamination with host derived EVs or proteins) of the starting material is also a significant issue when isolating parasitic helminth‐derived EVs from complex host matrices such as blood or intestinal contents instead of in vitro cultures. As helminth EVs are derived from the whole organism (containing diverse cells, tissues, and organs), they are likely to be heterogeneous in nature; this presents challenges in studying EV release from single cell‐types in these organisms (Figure 2). Finally, even when in vitro culture is possible, potential changes to EV production or cargo molecules can occur when helminths are taken out of their in vivo environment; the impact of this is difficult to assess. These issues must be considered when deciding how to separate, purify and quality control (QC) EV preparations for descriptive or functional studies.

**FIGURE 3:**
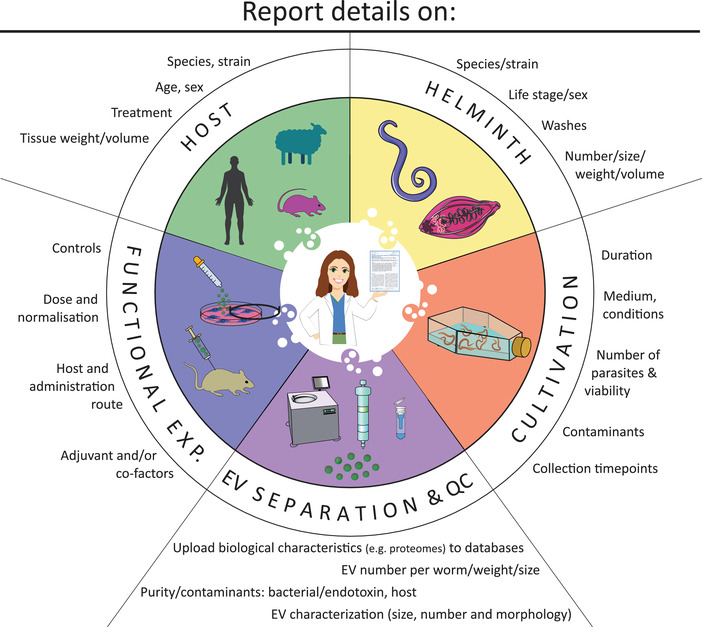
Community‐agreed details for harmonising the reporting of studies involving parasitic helminth EVs. Minimal information to be included in experimental reporting when working with parasitic helminth EVs in addition to the details specified in MISEV 2018 is summarised. These involve information about the host, where the helminths were collected, helminth specifications, culture conditions, EV separation & quality control (QC) and aspects to be included when performing functional experiments (further detailed in Textbox 2). The suggested standard for reporting these criteria is defined in Table 1. Part of the figure was created with adjusted images from Servier Medical Art, provided by Servier, licensed under a Creative Commons Attribution 3.0 unported license.

Despite the increasing numbers of investigators studying helminth‐derived EVs, there are no current standardised or unified methods for their isolation and characterisation that complement the recommendations proposed by the International Society for Extracellular Vesicles for the characterisation of mammalian and other eukaryotic EVs (Théry et al., 2018). Accordingly, the principal purpose of this position paper is to recommend guidelines for researchers and the wider community (i.e., journal editors and reviewers) on agreeable standards for studying helminth‐derived EVs. The secondary goal of this community‐contributed roadmap is to provide suggested protocols and best‐practice recommendations (Table 1, Figure 3) for unifying the future reporting of these difficult‐to‐study participants in interspecies communication. We anticipate that this roadmap could also provide a framework for researchers studying other pathogens.

**TABLE 1:** Guidelines for reporting helminth EV research

Criteria for reporting	Suggested standard
Helminths	
Helminth species including strain, genotype, drug specificity if applicable	Mandatory
Lifestage of helminth	Mandatory
Number, weight, sex and/or size or volume of worms	Mandatory if applicable
Washing buffer or media used (and number of washes)	Mandatory
Host	
Host species (and strain) used and origin when not a laboratory reared host (field isolates)	Mandatory
Host age and sex	Mandatory
Host tissue from where the helminth is isolated (Blood, faeces, etc) and tissue weight	Mandatory
Treatment if any	Encouraged
Number of host animals used	Encouraged
Helminth cultivation	
Culture length	Mandatory
Composition of culture media	Mandatory
Volume of culture media	Encouraged
Number of worms per ml of media	Mandatory if applicable
Incubation temperature and % CO2	Mandatory
Parasite viability ‐ description of viability assay performed (e.g., motility assay, visual inspection)	Mandatory if applicable
Number of parasite pre‐incubation washes and time	Mandatory
Depletion of culture media of EVs if applicable (e.g., FBS used)	Encouraged
Biological contamination test (mycoplasma, LPS, ES agar plating, other)	Encouraged
EV separation and QC	
ES collection timepoints and storage	Mandatory
EV storage details	Mandatory
EV separation technique used and details (details for reporting described in main text for each technique)	Mandatory
Details of low speed differential centrifugation and/or filtration performed to pre‐clear ES of eggs and debris	Mandatory
Upload of data in Vesiclepedia and other community‐based open source repositories (PRIDE for proteomics data)	Encouraged
EVs number per worm, weight or size	Encouraged
Reporting common residual host contaminants (i.e., host‐albumin)	Encouraged
Vesicle purity	Encouraged
Functional studies of helminth EVS	
Measurement of number of particles and /or size distribution and/or visualisation technique used (TEM, cryo‐TEM)	Mandatory if applicable
Protein/lipid/glycan quantification	Mandatory if applicable
Proteomic analysis of specific EV or EV‐enriched markers if available	Encouraged
Nucleic acid assessment and description of method used	Encouraged
EV labelling method	Mandatory
Controls of EV labelling and how they were obtained	Mandatory
Controls used for functional studies and how they were obtained	Mandatory
Normalisation of EVs for functional studies (i.e., number of vesicles/ml, number vesicles/worm)	Mandatory

*Note*: Criteria to be reported during publication of helminth EV research and suggested standard for requirement to report based on survey of authors.

Abbreviations: cryo‐TEM, cryogenic transmission electron microscopy; ES, excretory/secretory; EVs, extracellular vesicles; FBS, fetal bovine serum; LPS, lipopolysaccharide; TEM, transmission electron microscopy

Several aspects are addressed for consideration: (1) in vitro culture conditions and assessment of helminth viability for the purposes of collecting excretory/secretory (E/S) molecules and EVs; (2) isolation of helminth EVs from in vitro‐derived E/S or in vivo‐derived host tissues and organs; (3) QC and quantification of helminth EVs; and (4) considerations for in vitro and in vivo functional studies (Figure 1). Additionally, we provide here a specific reporting template (Table 1, Table S1, Figure 3) in order to facilitate standardisation across studies. These guidelines are not intended to be strict requirements; since helminths are a diverse group of organisms, not all of the recommendations will be applicable to all species. Instead, we hope for a workable set of standards to improve the quality and reproducibility of publications in the helminth EV field. We envisage that many of these aspects, and the obstacles associated with them, will also be relevant to other parasites, commensals and symbionts. Importantly, while we recognise the inherent variability within the helminth EV field may not allow researchers to meet all of the guidelines, we feel the most important recommendations are the reporting of sufficient methodological details at each stage (within the manuscript and to EV‐TRACK) and the submission of EV properties (e.g., proteomic, metabolomic, glycomic, mRNA, small RNA (sRNA), DNA, and lipid characterisation) to Vesiclepedia (Pathan et al., 2019) or other open access EV‐relevant databases such as PRIDE (Perez‐Riverol et al., 2019), SRA (Leinonen et al., 2011) or Glycopost (Watanabe et al., 2021).

## 2 IN VITRO CULTURE CONDITIONS AND ASSESSMENT OF HELMINTH VIABILITY

The initial starting material for isolation of helminth EVs is the excretory and secretory products of the worms, usually referred to as the E/S products. This is similar to what other fields may define as conditioned media, although some other biological material such as vesicular fluids from cestodes might also serve as a source of EVs. To simplify, we refer here to E/S products as all excreted/secreted molecules, including proteins, lipids, nucleic acids and vesicular material. E/S products are generated by maintaining the parasite(s) in media for a defined length of time, followed by collection of the resulting E/S. After collection, a series of steps can be used to filter out unwanted fractions from E/S, but the initial conditions for collecting E/S are important variables to consider and report. Differences in in vitro cultivation of helminths, such as the extent of washing to remove host material or intestinal microbiota may influence the purity of helminth derived EVs. Additionally, different parasite life stages, sexes, and the length of time in maintenance could produce different populations of EVs, affecting cargo description and functionality.

Although the cultivation of different helminth species is usually well‐standardised in terms of preferred medium, different laboratories might have their own specific method that may not be easily modified without compromising worm survival. To a degree, the methodologies for in vitro maintenance may dictate approaches taken for collecting the E/S prior to EV separation/purification. Steps that should be reported include the parasite species/strain/isolate examined, the sex being studied for dioecious helminths, the number, duration, and volume of washing steps (including composition of wash buffer) for helminths isolated from animal tissues or organs, the maintenance conditions, the length of time in maintenance, intervals at which E/S is collected if multiple collections are done during culture, and any tests for contamination (e.g., *Limulus* Amebocyte Lysate test for lipopolysaccharide contamination or *Mycoplasma* testing).

Details of helminth species should include the strain or genotype, provenance of strain, drug susceptibility (if known), how the species was molecularly/phenotypically identified (if not derived from a lab‐reared strain) and the host species strain, source and diet if applicable (i.e., in vaccination and microbiome studies). Furthermore, the number of animals used for worm collection, the duration of infection and developmental stage of the parasite should also be recorded. Reporting the number of parasites used for E/S collection will vary greatly based on the species studied, nevertheless, it should be justified. For example, the reporting of either the number and size, weight or volume of some developmental stages of cestodes (i.e., metacestodes) and trematodes is feasible, while, for some nematode species, reporting approximate numbers of individuals may be more practical. For all species, the culture volume should be reported, as well as incubator temperature, humidity and CO_2_ level. When parasite inner fluid is used for EV preparations (e.g., “metacestode vesicle fluid” in cestodes or “body fluid” in adult nematodes), the volume and methodology used to recover inner fluid should be reported. It is advised to keep the duration of parasite incubations as short as possible to limit parasite and cell death in the culture. Parasite viability during culture can be assessed through different assays (visual inspection, motility, ATP assays, viability dyes, etc.) and should be documented according to previously‐published methodologies (Guidi et al., 2020; Nguyen et al., 2021; Nowacki et al., 2015; Peak et al., 2010; Ramirez et al., 2007).

Biological contamination of E/S is not uncommon as many helminths are obtained from host tissues and organs, including the intestine. Washing helminths recovered from host tissues, organs or gastrointestinal tracts is common practice before parasite incubation in vitro, but this is often not adequately detailed in publications. The number, duration, and volume of washes, the media used for washing and any additional measures such as use of antibiotics (and concentrations) should be reported. However, contamination can still occur despite thorough washing of worms before in vitro culture and maintenance of worms in antibiotic/antimycotic‐containing media. Thus, it is encouraged that further measures should be routinely implemented to ensure E/S preparations are not contaminated by bacterial or host contaminants. For instance, common bacterial contaminants can be detected by performing *Mycoplasma* testing, endotoxin testing or assaying for bacterial growth (Borup et al., 2022; Ridolfi et al., 2020). Furthermore, any procedures designed to reduce contamination should also be noted (e.g., if media from the first several hours of culture are excluded from analyses or if (and how) host cells have been removed). Similarly, monitoring host or feeder cell contamination can be assessed by measuring the presence of an abundant host protein or host‐specific RNA by western blot or qRT‐PCR. It is possible, however, that in some models, specific host factors could be a biologically‐relevant component of helminth EVs. For example, parasites ingest and release host material naturally and it is possible that proteins (including those derived from the host) could associate with the surface of EVs, as part of a protein “corona” (Figure 2) as recently discovered for mammalian EVs (Tóth et al., 2021). Also, one report suggests that ∼40% of the miRNAs found in *T. muris* EVs derive from the host (White et al., 2020). Therefore, as this field evolves, rigorous and consistent documentation of all washing and QC steps will be essential to disentangle contamination from genuine signal in terms of specific host factor association with helminth EVs; this also applies to glycosylation (detailed further below).

Culture media should be reported including all additional components such as antibiotics or supplements. Fetal Bovine Serum (FBS) and any other components that can introduce exogenous EV sources should be avoided completely unless required for parasite survival. If so, an EV‐depletion (i.e., 18‐h ultracentrifugation at 120,000 *g*) step should be performed to remove exogenous EVs (Shelke et al., 2014) for recommended methods. However, no single method allows for the complete removal of EVs from FBS; therefore, it is of particular interest to include this information. If feeder cells are required to generate media for worms, then removing EV contamination from these feeder cell cultures should be described and evidence of sufficient depletion reported.

## 3 EV DEFINITION, SEPARATION AND CONCENTRATION

Helminth EVs, like mammalian EVs, can be diverse and the MISEV 2018 guidance given for the nomenclature and definition of EVs is also applicable to the helminth EV field. For species where no known EV protein marker is available, which will be the majority of helminths, EV size or density properties can be used to define EV populations. Small EVs (sEV) are less than 200 nm and medium or large EVs (m/lEVs) are greater than 200 nm. While limited number of studies have employed density gradients to define helminth EV populations (Chaiyadet et al., 2022; Chow et al., 2019; Gasan et al., 2021; Sotillo et al., 2016), in time, the mammalian EV nomenclature of low, middle or high‐density EVs will also likely be applicable to helminths.

In all areas of EV research, the trade‐off between purity and yield when separating and concentrating EVs is a challenge (more information about differences in yield/purity of the different EV isolation techniques can be obtained elsewhere (Théry et al., 2018)). Prioritising one or another based on the research question may influence the choice of methods used. This challenge is amplified in the helminth EV field where worms are obtained from living animals and incubated in vitro. It can be a time‐consuming process to obtain worms of a given developmental stage when the number of worms in each host is limited. This, in turn, could require the use of many experimental animals, which presents economic constraints and introduces ethical considerations (e.g., 3Rs; reduction, refinement and replacement of animals in scientific research). Additionally, the short duration of in vitro parasite maintenance can limit the amount of E/S generated.

To date, the majority of helminth EV research has utilised ultracentrifugation (UC) of E/S products as the predominant separation and isolation method, following the same trend seen across all EV research (Sotillo et al., 2020). However, as in the mammalian field, more recent research on helminth EVs has seen a shift towards using other methodologies such as size‐exclusion chromatography (SEC), iodixanol density gradient, ultrafiltration, or combinatorial approaches (Bennett et al., 2022; Chaiyadet et al., 2022; Gasan et al., 2021; Kuipers et al., 2022; Sotillo et al., 2020). Some methods will be impractical for the field at this time (i.e., immunoaffinity‐based techniques) since there is still no helminth‐specific EV biomarkers identified for each phylum. Nevertheless, and when helminth‐specific requirements are not required, MISEV 2018 guidelines for EV separation including the combination of different physical separation techniques should be considered.

Our guidelines do not recommend one particular method or combination of methods as standard practice for the isolation of parasitic helminth EVs (MISEV 2018 recommendations should be followed), but underscore the point that different methods will affect number, purity and types of EVs enriched and should be carefully detailed (Davis et al., 2019).

Storage conditions should be described for both E/S material prior to use for EV preparation, unless used fresh, and the EV preparation itself. Storage buffer, carrier proteins or cryo‐protectant, temperature and length of storage should be reported as well as the number of freeze thaw cycles. Furthermore, any processing of the collected E/S before (and after) EV isolation, such as differential centrifugation at slow speed, or filtering, to remove eggs or parasite debris, and methods of supernatant transfer, should be reported (Galiano et al., 2020).

## 4 CHARACTERISATION AND QUALITY CONTROL (QC) FOR HELMINTH EVs

Similarly to MISEV 2018 recommendations, it is desirable that more than one method is used for QC, such as nanoparticle size analysis, visualisation methods, western blots using specific antibodies, total lipid quantification (Osteikoetxea et al., 2015; Visnovitz et al., 2019), protein, glycan and/or RNA analyses.

Nanoparticle concentration should be reported in line with the recommendations published by MISEV in 2018 and any updated guidelines following improvements in detection sensitivities. In line with the recommendations from the ISEV Rigor and Standardization EV Reference Material Task Force (Welsh et al., 2020), the detection limit of the technique used should also be reported. Furthermore, it is specifically recommended to report the number of EVs in relation to the number/weight/size of the worms. Although this might not be feasible for small helminths, or some small immature lifecycle stages, it can be a useful metric for documenting the variabilities in EV production and secretion associated with different helminth species and for biological interpretation in functional studies.

### 4.1 EV‐associated proteins

Unlike many mammalian species, the helminth EV field has very few well‐characterised EV‐associated protein markers and, even when an EV‐associated protein has been identified, commercially available antibodies might not exist. In this regard, as is the case for mammalian EVs, an EV marker is expected to be enriched in EVs compared to parent cell lysates, and, in the specific case of helminths, enriched compared to the total E/S. As highlighted in the introduction, the term helminth refers to multiple, evolutionarily diverse species, and the helminth EV protein cargo and any helminth EV‐associated proteins reflect this diversity (Sotillo et al., 2020). Despite the identification of a pan‐helminth EV marker being unlikely, some common protein families are shared at least between some helminth species (Sotillo et al., 2020). Aside from species specificity, it should be noted that different EV preparation methods may bias specific subtypes of EVs and certain contaminating molecules. For example, proteomic analysis of *Fasciola hepatica* EVs isolated by different methods identified both overlapping and unique proteins (Davis et al., 2019; Murphy et al., 2020). Furthermore, in some helminth species, such as the blood and liver flukes or haematophagous worms, or life stages, it is especially relevant to determine the presence/absence of common blood contaminants such as albumin in EV preparations. Nevertheless, if a helminth‐specific EV protein marker is identified (within a species or a larger taxonomic grouping) and a way to detect it is available (e.g., commercial, cross‐reactive antibody), then this specific protein marker should be differentially quantified in EV‐enriched versus EV‐ depleted preparations derived from helminth E/S products.

### 4.2 EV‐associated glycosylation

A novel area of helminth research surrounds the identification of EV associated glycans, which could impact various aspects of cellular recognition/uptake as well as immune modulation that could be highly relevant to the larger EV field. As characterisation of glycosylation in helminth EVs is fairly new, and different species may carry unique glycans, defining the glycome of more helminth species EVs will be beneficial for future QC considerations. Additionally, during the analysis of helminth EV glycomes, methodologies must account for the presence of novel glycans as most helminth glycomes remain unknown and cannot be predicted based on the genome. Identifying and characterising species‐specific helminth EV‐associated (glyco) proteins is ongoing and will help to develop our understanding of EV preparations, as well helminth‐host interactions. In this regard, researchers are encouraged to perform full proteomic analysis of their EV population in order to better understand EV associated proteins present in the species of interest, and it is recommended that newly characterised protein markers and EV cargo are submitted to Vesiclepedia (http://microvesicles.org/) and/or EVpedia (http://evpedia.info/evpedia2_xe/) to allow for accessibility (Pathan et al., 2019). Methods such as enzymatic surface shaving of EVs have also been combined with proteomics or glycan analysis to help determine the localisation and role of different EV‐associated proteins or glycans (Allen et al., 2021; Cwiklinski et al., 2015; de la Torre‐Escudero et al., 2019; Kuipers et al., 2020; Wititkornkul et al., 2021). A brief summary of proteins that have been identified in descriptive studies of parasitic helminth EVs as potential pan‐phyla EV markers is provided (Textbox 1) with a comparative analysis of EV associated proteins across multiple helminth species described recently (Sotillo et al., 2020). We hope that future revisions of these guidelines will lead to the use of increasingly sensitive approaches to characterise helminth‐associated EV (glyco) protein cargo and host/culture contaminants in order to improve QC.

### 4.3 Assessment of nucleic acids

RNA is a ubiquitous cargo component of EVs across diverse organisms including helminths. However, the detection of nucleic acid cargo is not commonly used as a method of QC, primarily because there is less understanding of the secretion/packaging mechanisms to define which sequences are truly confirmed EV‐RNAs. Nevertheless, comparison of miRNA cargo across diverse helminths has identified some sequences which are consistently found, such as miR‐10 and let‐7, as well as other helminth‐specific miRNAs (Cucher et al., 2021; Sotillo et al., 2020; White et al., 2020). The interest in using EV‐derived miRNAs as diagnostic biomarkers in helminth infections has also been growing recently (Mu et al., 2021). Additionally, as further non‐vesicular extracellular RNA‐binding protein complexes are identified in E/S, it is becoming clear that not all extracellular RNAs are within EVs. One approach for testing whether RNA is present inside EVs is RNase sensitivity assays, where EV‐RNA can be identified through sensitivity to detergent (Buck et al., 2014). Some sRNA species such as Y‐RNAs are degraded when outside of EVs and detection of full‐length products can be used as a marker of intact EVs (Buck et al., 2014). However, these directed approaches do not always consider that other nucleic acids, outside of EVs, could also be functional. Likewise, nucleic acids on the outer membrane of vesicles cannot be ignored as these could also serve functional or structural roles. It should also be noted that different library preparation methods and enzymatic treatments can have a large effect on the nucleic acids that are detected in helminth EVs, which contain some classes of small RNA (secondary siRNAs) that are distinct from mammals (Sotillo et al., 2020; White et al., 2020). Thus, reporting library kits used and accounting for this when comparing across data from different laboratories is advised (Chow et al., 2019; White et al., 2020).

The sRNA cargo of helminth EVs has been a major focus in the helminth EV field and provides a potential mechanism for host modulation. A number of different sRNA biotypes have been described as components of helminth EV cargo with certain biotypes being more or less abundant in different species or life cycle stages examined (Cucher et al., 2021). However, in mammalian systems, EVs can also contain other RNAs such as long non‐coding RNAs (lcRNA), circular RNAs (circRNA), and full‐length or fragmented messenger RNA (mRNA) (Kim et al., 2017). So far, full length mRNA has only been described from EVs in one helminth species, *Trichuris muris*, and we have yet to understand whether translation of a parasite mRNA can occur within the host cell, although it is an exciting possibility (Eichenberger et al., 2018b). Mammalian EVs have recently been shown to cargo fragmented genomic or mitochondrial DNA either within EVs, mostly in larger sub‐populations such as microvesicles and apoptotic bodies, or on the outer EV membrane in smaller EVs (<200 nm) (Malkin & Bratman, 2020). It seems likely that these nucleic acid biotypes will be explored in helminth EVs and reporting of findings to Vesiclepedia is encouraged.

### 4.4 Determining purity

The issue of EV purity is complex and, as noted above, quantification of proteins will be influenced by both canonical EV proteins, EV corona proteins and non‐EV associated contaminating (glyco) proteins. It is important to emphasise the need for a procedural control to determine background levels of particles that are detected by the instrument (for example, a control might be a non‐conditioned medium or EV depleted E/S that has gone through the same processing steps as a real EV sample preparation). One proposed measure of purity is to determine the ratio of particle number to protein concentration as proposed by Webber and Clayton (2013). This is widely used in the mammalian EV field and could be adapted by the helminth EV community. It is worth mentioning, possibly due to the difficulty in obtaining large amounts of EVs from most helminth species as detailed above, ratios observed will be lower than those described for mammalian EVs (Eichenberger et al., 2018b; Sotillo et al., 2016). Therefore, this ratio will need to be standardised for the different helminth species or life stage studied. Although non‐aggregated protein contaminants may not alter particle measurements, there is still the possibility that other non‐EV particles (e.g., protein aggregates) can be present which share similar physical properties with EVs and may be included in particle measurements. This factor needs to be considered when discussing purity of parasitic helminth‐derived EVs.

## 5 CONSIDERATIONS FOR FUNCTIONAL STUDIES IN VITRO VERSUS IN VIVO

### 5.1 Labelling of EVs

For studies related to the uptake of labelled EVs, specific labelling guidelines recommended by MISEV 2018 and future updates should be followed. Specifically for helminth EV research, an appreciation for the background level of dye remaining after clean up should also be evaluated by labelling an EV free buffer, or EV‐depleted E/S, using the same methodology. If the dye is specifically incorporated into the isolated EVs, or non‐EV associated dye can be adequately removed to the same level as a media control that is not exposed to helminths, this ‘in vivo*’* labelling method reduces the amount of off‐target labelling EV manipulation during processing (Boysen et al., 2020).

### 5.2 Controls

Pinpointing the direct functional effects of EVs is challenging both in vitro and in vivo. Therefore, it is important to carefully consider which controls to include when studying helminth‐derived EVs. To demonstrate that observed effects are associated with EVs rather than with soluble molecules, suggested controls to consider would be the helminth E/S products as well as EV‐depleted E/S. This also builds context for whether functional effects are unique to EVs, or shared with other non‐vesicular components of the E/S. When used, a detailed explanation of preparation and storage for this EV‐depleted E/S should be included along with validation of adequate EV depletion.

It is also recommended to include an appropriate mock negative control that has undergone similar treatments as EV isolation procedures, such as PBS, plant‐derived vesicles (e.g., grape‐derived EVs), target cell‐derived EVs or commercial EVs (Eichenberger et al., 2018a). Additional controls to consider when studying uptake are the use of helminth EV‐specific antibodies, or internalisation‐blocking compounds, which will indiscriminately block EV uptake (Chaiyadet et al., 2022; Coakley et al., 2017). Furthermore, conducting internalisation experiments at different temperatures (4°C vs. 37°C) or using methods to damage EVs (e.g., freeze‐thawing, sonication or UV treatment) could also provide suitable controls (Cheng et al., 2019; Eichenberger et al., 2018a; Marcilla et al., 2012; Nizamudeen et al., 2021). For analysis of functional RNA, where possible, Proteinase + RNase treatment of EVs can be used to ensure non‐vesicular RNA is removed so that RNA mediated effects on host cells are mediated by intra‐vesicular RNAs and not extra‐vesicular RNA. At the same time, these treatments could damage the native structures of the EVs and new findings have shown that cell surface‐associated RNAs may have structural or functional roles (Flynn et al., 2021). Caution is merited, therefore, in the approach taken in each study, which should be justified. Since the dose and temperature of RNase treatment will influence the extent of degradation, these variables also need to be reported. Furthermore, researchers should consider/ensure whether any treatments (such as RNase), and subsequent inactivation, could damage EVs or influence functional experiments (e.g., contaminating RNases could impact uptake studies into host cells). Similarly, enzymatic removal of glycans can be utilised to assess their ability to bind host lectins and how this may limit their ability to function. However, when interpreting the results of ‘shaving’ glycans, one should consider if this could lead to exposure of other EV surface molecules that may not normally be exposed. Glycan interactions with host lectins may also be assessed using lectin arrays, however, further experiments with host cells to validate interactions in the context of infection should also be performed.

When performing experiments to address the function of helminth‐derived EVs, researchers should consider simulating the in vivo conditions, which may require additional controls. This consideration will clearly be different for different helminths and tissue sites that these helminths occupy within the infected hosts. Researchers should also consider recapitulating biological settings by choosing a biologically reasonable cell line or tissue (e.g., cholangiocytes for parasites residing in the bile ducts). Additionally, consideration for how EVs interact with these cells in vivo is encouraged; for example, delivery of EVs to the apical or basal side of intestinal organoids depending on parasite localisation. A summary of further considerations for functional studies is provided (Textbox 2).

### 5.3 Dose

It is important to consider physiologically relevant doses of EVs in experiments, but this is difficult to empirically determine. For example, calculating the number of EVs produced per worm may give an estimate of EV production during in vitro culture, but may not perfectly represent what is produced in an in vivo setting. Where possible, especially for in vitro studies, using dosage experiments to determine how dose may influence the effect is recommended (see “Normalisation” section for how to provide information in dosage experiments).

### 5.4 Normalisation of EVs

Depending on the specific question to be answered, different normalisation strategies to compare the effect of independent EV populations or the effect of EVs versus the total E/S can be used. In this regard, wherever possible, using two different and independent normalisation methods is recommended. Traditionally, the most commonly used normalisation approach in the helminth and mammalian fields is based on particle number (if a particle number analysis was performed); however, using this method alone has limitations (i.e., protein aggregates may have similar characteristics as EVs; see ‘Determining Purity’ section). In this regard, if available, normalisation could be performed using a helminth‐specific marker (either protein, lipid or nucleic acid). Due to the variabilities in co‐purifying proteins during the diverse EV isolation procedures, protein concentration should only be given as a supplement to EV number.

Difficulties arise when considering how to best approach the normalisation of E/S and EV‐depleted E/S that is comparable to the EV fraction. The concentration of E/S to be used as a control can be calculated based on the volume of E/S used for the isolation of EVs. For methods such as UC, this is straightforward, but not for methods like SEC where the EV‐depleted fractions are more poorly defined. It is worth noting that SEC also allows for the collection of soluble proteins from a particular sample, which could be used for normalisation purposes. Researchers should also take into account possible non‐helminth derived EVs in media (avoid FBS) or biological fluids and, where possible, take steps to deplete these fluids of EVs.

### 5.5 EVs for vaccination and immunomodulation studies

For immunological studies and all in vivo experiments, the species/strain of the host, age, sex and any genetic background (strain) and manipulation of both the host and/or the helminth should be reported. When using EVs as a vaccine candidate, it is important to stipulate the number and interval of doses used, the adjuvant used and the route of administration, as different adjuvants and routes of administration influence immune responses. If possible, the kinetic and spatial tracking of labelled helminth EVs in the host should be performed to provide important information regarding tissue tropism and cellular uptake. When studying the effect of EVs on the host immune response it is imperative, as with other in vitro and in vivo experiments, to perform endotoxin tests as contamination will influence the immune response and risk the death of an animal.

## 6 FUTURE PERSPECTIVES

One of the major limitations faced by helminth EV researchers is the lack of universal helminth EV biomarkers. Emphasis should be placed on describing the biological content (for example prioritising proteins or RNA species) of different EV populations derived from parasitic helminths. The lack of a universal biomarker also impacts other areas of research such as imaging and characterisation of the internalisation processes of EVs within cells. In this sense, the development of panels of recombinant EV proteins (and subsequent production of antibodies against these proteins) and gene silencing/editing tools such as CRISPR‐Cas9‐induced reporter gene expression will not only allow for the characterisation of these processes, but also the ability to track helminth EVs in vivo. Additionally, in the search for the physiological conditions in which EVs act as mediators of host‐parasite or parasite‐parasite cross‐communication, the detection and quantification of EVs in vivo can guide their use in in vitro experiments. While challenging, potential methods might include immunogold staining of TEM sections of infected tissues, matrix‐assisted laser desorption ionisation (MALDI) imaging or other proteomic techniques (e.g., spatial proteomics), detection of parasite‐specific EV‐RNA or glycans and the use of antibodies to affinity purify and quantify helminth EVs from the specific fluids of infected animals. Over the coming years, there may also be scope for incorporating labelling into the worms prior to infection, loading of helminth vesicles with reporter molecules and additional genetic approaches.

Web‐based databases developed to compile the information on RNAs, proteins, lipids and metabolites identified in EVs from eukaryotic and prokaryotic organisms such as Vesiclepedia are extremely useful (Pathan et al., 2019), including an initial score‐based QC modelled on EV‐TRACK assessment (Van Deun et al., 2017). However, since the field is rapidly growing, a helminth‐specific EV database is increasingly needed. Such a database will allow for the comparison of EV isolation methods from different helminths and for the detection of similarities and differences in EVs from different helminth species, life stages or EV preparation methods. This, in turn, will facilitate the identification of parasitic EV markers to be used for studies characterising the EVs and their function. Due to the heterogeneity in maintenance conditions and parasite species in the helminth‐EV field, also detailing the common contaminants and associated proteins that are identified during in vitro, and in vivo, conditions are also highly relevant.

Finally, to expand and encourage collaboration among researchers, disseminate recent research findings and discuss the challenges and opportunities associated with EV research in helminths, specific workshops could be implemented at different parasitology conferences such as the one organised as part of the Parasitic Helminths Conference held in Hydra, Greece in 2019. Similarly, monographic special issues published by parasitology and/or immunology journals such as the ones published by the International Journal for Parasitology, and Molecular Immunology will contribute in this direction (Hoffmann et al., 2020; Ramirez & Marcilla, 2021)

## 7 CONCLUSIONS

These guidelines aim to provide general recommendations for methodologies and reporting in the helminth EV field. As summarised in Figures 1, 2, 3, the major points addressed herein are:

1. Different helminths require specific maintenance conditions, which might also differ between laboratories, particularly as new technologies evolve. This makes standardisation of methodologies challenging when not described in detail. The source of parasites, their host, pre‐washing steps and cultivation methods, including media composition, duration of culture and viability of worms, should be sufficiently reported.
2. Similar to that proposed by MISEV 2018, the EV separation methodologies used (including E/S processing and EV isolation) must be reported in detail in order to allow replication by other laboratories.
3. One of the most critical challenges in the helminth EV field is the lack of appropriate protein markers for discriminating between EV populations. Effort should be made into characterising the proteomic composition of helminth EVs, EV‐depleted E/S, and potential contaminants. Furthermore, it is strongly recommended that EV purity be assessed by a combination of size distribution and visualisation techniques. As the field advances, lipid, glycan and nucleic acid characterisation could also enable discrimination and assess purity of helminth EV preparations.
4. An emphasis should be placed on using the appropriate controls for the demonstration of specific helminth EV functions. These controls might vary depending on the specific helminth studied and its life stage, although it is important to demonstrate that function is specific to EVs and not contaminants from the soluble E/S.

### 7.1 Textbox 1

Helminths are a heterogeneous group of parasitic and free‐living worms that include members from two predominant and dissimilar phyla, Platyhelminthes (flatworms) and Nematoda (roundworms). This diversity is reflected in their biology, lifecycle and the content of their excretory/secretory products. The disparity in the methodology employed for EV isolation and the differences between nematodes and flatworms, among other reasons, hinder the identification of a common EV marker such as those identified in mammalian organisms. A systematic analysis performed on the proteomic content of EVs secreted by all helminths characterised so far has shown that M13 metallopeptidases and actin may have potential as markers for nematodes, while proteins belonging to the EF‐hand family could serve as pan‐platyhelminth EV markers (Sotillo et al., 2020). Standardised reporting of helminth EV data as well as availability in public repositories for their reanalysis, will contribute to the identification of common proteins that might ultimately serve as species‐ or phylum‐specific biomarkers.

### 7.2 Textbox 2

A longer‐term goal and opportunity in helminth EV research is to understand the functional and therapeutic properties of EVs, since EVs mediate parasite‐host interactions and may have immune‐modulatory properties relevant to other diseases including allergy and autoimmunity. In this regard, rigorous design and reporting of experiments are important for researchers to build on one another's results and, ultimately, translation. It is encouraged that information related to vesicle dose, and purity is included when reporting. For example, the number of EVs used in experiments (ideally accounting for doses in terms of EVs/individual worm and/or EVs/ml of excretory/secretory products). It is also advisable to include as much information as possible on the ‘omics’ analyses that go alongside the functional studies (e.g., the proteomic, nucleic acid and lipid cargos that are detected or identified for the first time in the EVs). Ultimately, rigorous reporting of the cargo molecules is useful for characterising their functions but could also provide better biomarkers of EV classes in different models, which can also help refine purification methods.

## AUTHOR CONTRIBUTIONS

Ruby White and Javier Sotillo prepared the online surveys, analysed survey results (summarised in Table S1) and other input, drafted the manuscript/revisions and communicated with authors.  Amy H. Buck and Karl F. Hoffmann organised the community, acquired funding and, together with Peter Nejsum, supervised the assembly and completion of the revisions and associated material. Maja Kosanovic, Marije E. Kuipers and Alicia Rojas designed Figures 1, 2 and 3. Microscopic images, embedded in Figure 2, were provided by Russell M. Morphew, Hynek Mazanec, Amy H. Buck, Anders Toftegaard Boysen, Ruby White and Marije E. Kuipers. All other authors contributed in developing the narrative that represents this community‐agreed roadmap.  All authors reviewed and approved the final draft of the manuscript.

## CONFLICT OF INTEREST

We declare no conflicts of interest.

## Supporting information

Supplementary information
